# The topology of the bacterial co-conserved protein network and its implications for predicting protein function

**DOI:** 10.1186/1471-2164-9-313

**Published:** 2008-06-30

**Authors:** Anis Karimpour-Fard, Sonia M Leach, Lawrence E Hunter, Ryan T Gill

**Affiliations:** 1 Center for Computational Pharmacology, University of Colorado School of Medicine, Aurora, Colorado 80045, USA; 2 Department of Chemical and Biological Engineering, University of Colorado, Boulder, CO 80309, USA; 3 Department of Electrical Engineering (ESAT), Research Division SCD, Katholieke Universiteit Leuven, B-3001 Leuven, Belgium

## Abstract

**Background:**

Protein-protein interactions networks are most often generated from physical protein-protein interaction data. Co-conservation, also known as phylogenetic profiles, is an alternative source of information for generating protein interaction networks. Co-conservation methods generate interaction networks among proteins that are gained or lost together through evolution. Co-conservation is a particularly useful technique in the compact bacteria genomes. Prior studies in yeast suggest that the topology of protein-protein interaction networks generated from physical interaction assays can offer important insight into protein function. Here, we hypothesize that in bacteria, the topology of protein interaction networks derived via co-conservation information could similarly improve methods for predicting protein function. Since the topology of bacteria co-conservation protein-protein interaction networks has not previously been studied in depth, we first perform such an analysis for co-conservation networks in *E. coli *K12. Next, we demonstrate one way in which network connectivity measures and global and local function distribution can be exploited to predict protein function for previously uncharacterized proteins.

**Results:**

Our results showed, like most biological networks, our bacteria co-conserved protein-protein interaction networks had scale-free topologies. Our results indicated that some properties of the physical yeast interaction network hold in our bacteria co-conservation networks, such as high connectivity for essential proteins. However, the high connectivity among protein complexes in the yeast physical network was not seen in the co-conservation network which uses all bacteria as the reference set. We found that the distribution of node connectivity varied by functional category and could be informative for function prediction. By integrating of functional information from different annotation sources and using the network topology, we were able to infer function for uncharacterized proteins.

**Conclusion:**

Interactions networks based on co-conservation can contain information distinct from networks based on physical or other interaction types. Our study has shown co-conservation based networks to exhibit a scale free topology, as expected for biological networks. We also revealed ways that connectivity in our networks can be informative for the functional characterization of proteins.

## Background

Co-conservation, a measure of the degree to which proteins are gained and lost together through evolution (also known as a phylogenetic profile [1]), has demonstrated utility as a protein function prediction method [2-13], particularly in bacteria. Pairwise co-conservation scores can be aggregated into networks [7], and assessments of connectivity within the resulting graph can further improve the quality of function prediction. Function prediction methods based on biological networks is an active area of research [14].

Topological analysis of other types of biological networks, including protein-protein interactions, regulatory interactions, and metabolic networks, has demonstrated that structural features of network subgraphs can provide quantitative insight into biological function [15-33]. For example, Maslov and Sneppen analyzed the stability of interaction networks by comparing patterns in average connectivity of interaction and regulatory networks [26]. Characterizations of the structural features of metabolite networks [15,16,20,25,29-31] demonstrate a correlation between topologically defined subnetworks and biochemical function. Topological characterizations also illuminate evolutionary issues. For example, Fraser et al. observed that the effect of an individual protein on cell fitness correlates with the number of its interaction partners [23]. Jeong et al. showed that most highly connected proteins in protein-protein interaction networks are crucial to cell viability [24].

Due to the availability of genome-wide data, nearly all previous investigations of network topology have been in yeast, and the majority has been based on high-throughput assays of protein-protein interactions (PPI). In contrast, this paper examines co-conservation networks in bacteria using different reference genomes, the first in-depth study to our knowledge, of the topological characteristics of such networks. This characterization can be used for current and future comparison to like studies in other organism and network types. Co-conservation networks are distinct from physical interaction networks as they capture putative functional relationships which are not necessarily dependent on direct protein binding. We find that bacterial co-conservation networks show both biologically important similarities and differences with yeast PPI networks. For example, similar to reports of significance in yeast PPI networks [24,34,35], node degree (the number of other proteins that a protein is connected to) in bacterial co-conservation networks is predictive of broad functional categories, such as essentiality. Unlike yeast PPI networks [26,28,34], the bacterial co-conservation network using all bacteria as the reference set does not demonstrate high connectivity among proteins that form complexes.

Such differences call into question the broad applicability of the yeast methods for predicting function based on network topology. Here, the topological properties of bacterial co-conservation networks and their relationship to function are examined. Based on this assessment, we demonstrate the use of co-conservation network topological properties to predict the function of uncharacterized proteins.

## Results and discussion

**Figure 1:**
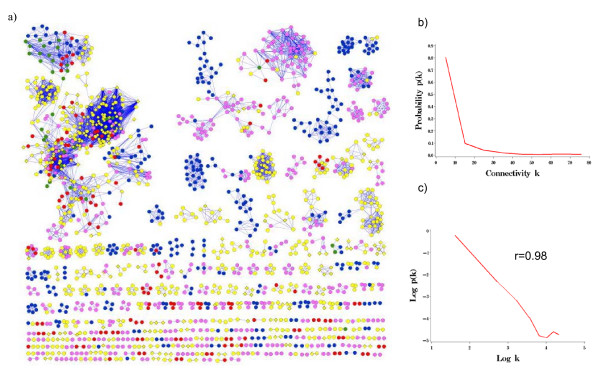
**a) Co-conserved protein-protein interaction network of *E. coli *K12 using All bacteria as the reference genome set.** Nodes are colored based on KEGG functional category. Note co-conservation based interactions occur between proteins that function in related processes. Metabolism (pink); Genetic Information Processing (red); Environmental Information Processing (blue); Cellular Processes (green); Unclassified (yellow); Unclassified in KEGG, COG and TIGR (diamond); b) Connectivity distribution of co-conserved protein-protein interactions: b) connectivity (k) versus p(k); c) log connectivity (k) versus log p(k).

The bacterial co-conservation network constructed from *E. coli *K12 and 267 other completely sequenced bacteria (referred to as the All reference set) is shown in Figure 1a. This network captures, for each *E. coli *K12 protein, which other proteins it is co-conserved with in a large reference group. We have previously described how such interaction networks can vary with the selection of reference group [8] and that networks conserved across multiple species can provide insight into the function of proteins that act in coherent biological processes [7]. Here, we assessed the topological characteristics of bacterial co-conservation networks for the purpose of using such characteristics to improve protein function prediction. Though most results are reported using the All genomes reference set, we also provide comparison to using Motile, Proteobacteria and Aerobic reference sets.

### Topological features of *E. coli *K12 networks

The *E. coli *K12 co-conservation network using all genomes as reference (the All network) contained 6,987 interactions, among 1,700 proteins, forming 312 disconnected subgraphs, called clusters (Table 1). The remaining 2,537 *E. coli *K12 proteins were not co-conserved with any others, and would be singletons if they had been included in the graph. Three large clusters were apparent, containing 417 proteins (with 4,157 interactions), 80 proteins (with 203 interactions) and 58 proteins (with 353 interactions). Surprisingly, networks constructed from the other reference sets (Motile, Proteobacteria, and Aerobic) contained roughly the same number of proteins as the All network, yet the number of interactions varied widely (Table 1).

**Table 1:** Topological analysis of the networks.

	All	Motile	Proteo	Aerobic
Number of interactions (edges)	6,987	16,905	24,047	3,825
Number of proteins (nodes)	1,700	1,990	2,072	1,410
Log-log correlation (r)	0.98	0.96	0.91	0.88
Power law exponent (γ)	1.77	1.6	1.38	2.21
Average clustering coefficient (c)	0.81	0.74	0.75	0.77
Connectivity average (k)	8.22	16.98	23.21	5.42
Standard deviation of connectivity	12.96	27.22	42.94	5.42
Average shortest path	5.11	8.40	4.98	5.96
Diameter	14	25	21	17

The clustering coefficient was defined as the edge density in the neighbors of a protein. The average clustering coefficient of the All network was high (0.81), indicating that proteins tend to be co-conserved in highly connected groups. The average shortest path (5.11) indicated that there was a short path between any two proteins in a cluster. The average clustering coefficient remained high for Motile, Proteobacteria, and Aerobic networks. Though the connectivity average increased as the number of interactions increased, the average shortest path appeared to be large in the Motile network, relative to the others. Moreover, the diameter of the Motile network was disproportionately large. This occurred because the Motile network consisted of two large densely connected clusters bridged by only a few edges and there existed many smaller clusters extending by long paths from the two dense cores (Additional file 1).

The connectivity distribution P(k), the probability that a protein interacts with k other proteins, shown in Figure 1b for the All network, had a heterogeneous, skewed shape, and indicated that most proteins were linked to only a few proteins, but a few proteins had a large number of connections. P(k) in this graph was consistent with a power law distribution P(k) ~ k^-γ^, with γ = 1.77 (Figure 1b), indicating a scale free network. In the log-log plot of Figure 1c, there was a high correlation (r = 0.98) between connectivity (k) and connectivity distribution (P(k)) for a large range of k. However the correlation broke down for highly connected nodes. Topological analysis is summarized in Table 1 using different reference sets. Like many other biological networks [22,25,31], the bacterial co-conservation networks using difference reference sets were all scale-free (Additional file 1).

#### Hubs in scale free networks distinguish essentiality and complexes

Scale-free networks share a number of properties, including sensitivity to disruptions of highly connected "hub" nodes and robustness to interference with non-hub nodes, among others. Therefore, this result suggests that the collection of hub nodes in bacterial co-conservation networks should include a higher proportion of essential proteins than the collection of non-hub nodes. Indeed, essential proteins in our bacterial co-conservation network had significantly more interactions than non-essential proteins (see Table 2, except Proteobacteria), validating earlier findings in yeast using other types of interactions [24,35,36]. For example, the average degree of essential proteins in the All network was 15.77 (SD = 19.93), while that of non-essential proteins was 7.87 (SD = 12.49) (p = 0.0003). Note that the disparity of mean connectivity between essential and non-essential proteins suggests that connectivity alone can be used as a proxy for essentiality. In the Proteobacteria network, the essentiality is not predictable (Table 2). In that essentiality is not evenly distributed among functional categories, these results suggest that connectivity, and possibly other measures of network topology, might also be useful for improving function prediction.

**Table 2:** Connectivity of essential versus non-essential and complex versus non-complex in the co-conserved protein-protein interaction networks.

	Essential/non-essential				Complex/non-complex			
	All	Motile	Proteo	Aerobic	All	Motile	Proteo	Aerobic
Mean connectivity	15.77/7.87 (p = 0.0003)	25.97/16.56 (p < 0.0001)	25.96/23.12 (p = 0.41)	7.48/5.33 (p = 0.025)	7.15/8.73 (p = 0.4)	12.66/18.73 (p = 0.0005)	13.21/26.77 (p = 0.03)	5.96/5.17 (p = 0.001)
Std connectivity	19.93/12.49	31.02/27.54	45.89/42.86	7.30/5.31	9.10/14.13	20.76/29.97	24.05/47.41	5.36/5.44
Max connectivity	71/81	135/147	199/199	35/35	77/81	142/147	160/199	35/35

Unlike yeast PPI networks [26,28,34], high connectivity in bacteria co-conservation networks does not allow identification of protein complexes. We noted that when the reference genome was All, there was no significant difference between the connectivity of complex proteins vs. non-complex proteins (Table 2). The small difference between mean connectivity in the Aerobic network is statistically significant due to low overall standard deviation of connectivity (Table 1). Interestingly, high connectivity in the Motile and Proteobacteria networks corresponds to non-complex proteins. This is in contrast to yeast PPI networks where high connectivity corresponds to protein complexes.

### Relationship between protein-protein interaction and co-conservation networks

The co-conservation network was compared to the *E. coli *K12 protein-protein interaction (PPI) network, obtained from the Database of Interacting Proteins (DIP) [37], which contained 4,922 interactions over 1,266 proteins. The PPI network had no interactions for 1,144 proteins that had at least one interaction in the co-conservation network (the All network). Of these 1,144 proteins in the co-conservation network, 533 were unclassified using KEGG (42% have at least 1 annotated neighbor), 182 were unclassified using COG (80% with an annotated neighbor) and 460 were unclassified using TIGR (57% with an annotated neighbor). These proteins represent examples where the co-conservation network allowed the assignment of function to proteins which could not be annotated using the PPI network.

### Relationships among topological characteristics and protein function

#### Function annotation homogeneity within clusters

**Figure 2:**
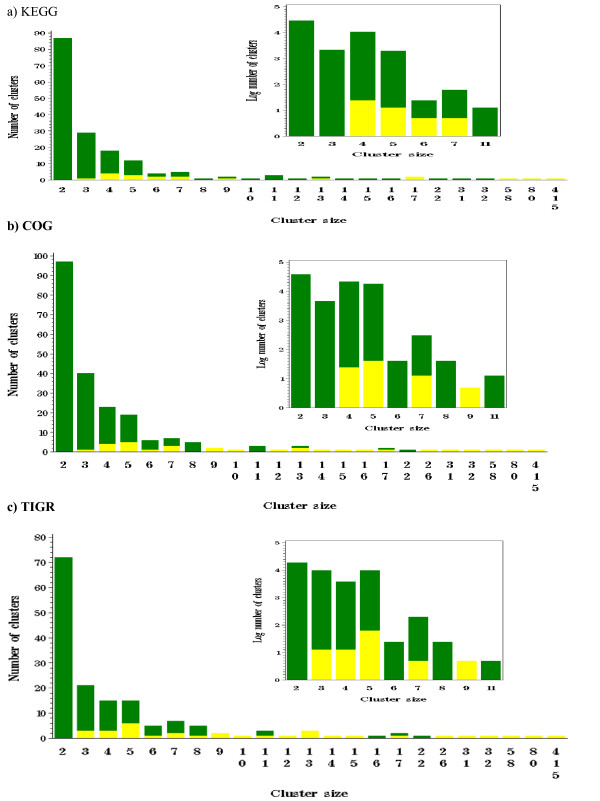
**The relationship between cluster size and function assignment.** For clusters with at least two classified proteins, the number of clusters where *all *classified proteins in the cluster share the same functional category (green) or different categories (yellow). Unclassified proteins were not considered in the comparison. The x axis is the cluster size and the vertical bar shows the numbers of clusters. The insets show this information on the log scale for a cluster size <= 11. a) KEGG b) COG c) TIGR.

The co-conservation protein clusters in the All network ranged in size from 2 to 417 proteins. Considering clusters with at least two annotated proteins, the proteins in all but the largest clusters tended to have identical function annotations, regardless of the source of annotation (Figure 2). For clusters of size less than 11, the majority are homogeneous for all different sources. Though some medium and large clusters contained proteins with different function annotations, these proteins are often involved in inter-dependent processes that contribute to a common phenomenon [8]. For example, many clusters contain proteins annotated to some specific function and also to "regulation," where the regulation activity turns out to be regulating the process in which the other proteins participate. The proteins that underlie a function and its regulation were co-conserved. This was not an indication of functional heterogeneity, but was an artifact of the annotation scheme.

#### Function and connectivity

**Figure 3:**
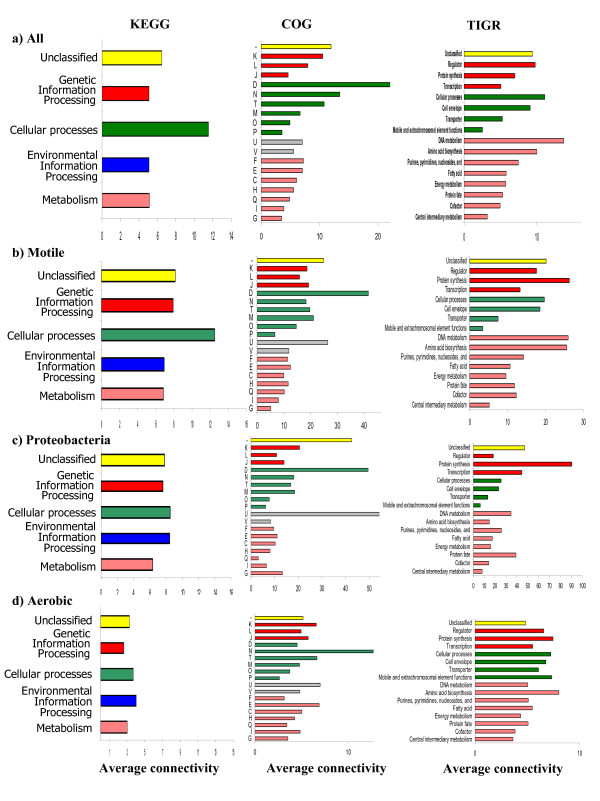
**Average connectivity per functional category, using different reference sets and annotation sources.** a) All b) Motile c) Proteobacteria d) Aerobic. COG functional categories and subcategories are: Poorly characterized [Not classified (-)]; Information storage and processing [Translation, ribosomal structure and biogenesis (J); Transcription (K); DNA replication, recombination and repair(L)]; Cellular processes [Cell division and chromosome partitioning (D); Posttranslational modification, protein turnover, chaperones (O); Cell envelope biogenesis, outer membrane (M); Cell motility and secretion (N); Inorganic ion transport and metabolism(P); Signal transduction mechanism (T); Intracellular trafficking, secretion, and vesicular transport (U); Defense mechanisms (V)]; Metabolism [Energy production and conversion (C); Carbohydrate transport and metabolism (G); Amino acid transport and metabolism (E); Nucleotide transport and metabolism (F); Coenzyme metabolism (I); Lipid metabolism (H); Secondary metabolites biosynthesis, transport and catabolism (Q)].

Different functional classes displayed different average connectivity (Figure 3). Since the definition of a functional class varied among different annotation databases, Figure 3 shows the connectivity of each protein versus KEGG [38], COG [39], and TIGR [40] functional categories. For KEGG and COG, regardless of the reference set, proteins with high connectivity were generally involved in cellular processes (Figure 3, green bars), which was interesting given that there were few cellular process proteins in each network. In contrast, metabolism proteins (pink bars) were abundant and generally had the lowest average connectivity.

Using COG, cell division and chromosome partitioning (D) in Figure 3 had high average connectivity using all reference sets except Aerobic. In the Proteobacteria network, the most highly connected protein were intracellular trafficking and secretion (U) while the most highly connected proteins in the Aerobic network were motility proteins (N). TIGR classification showed proteins involved in Motility had high average connectivity, while Central intermediary metabolism and Cofactors were the least connected (Figure 3).

Translation is one of the most ancient processes in the cell and previous studies have shown that these proteins have a high average connectivity [27,41]. The average connectivity of these nodes in our networks was low (J in COG and Protein synthesis in TIGR, Figure 3); this was because most of these proteins appeared in more than 90% of organisms and were removed in the preprocessing step as described in the Methods section. Additional file 2, 3, 4 show the presence of high connectivity among these proteins in networks when proteins that appear in more than 90% or less than 10% of the genomes in a reference set were not removed. Interestingly, Przulj et al. have earlier observed that in the yeast protein-protein interaction network, stress and defense and transport proteins are less connected than transcription and translation proteins [28]. Though the annotation sources for *E. coli *are completely different, the average connectivity in TIGR for transporter and transcription were similar (Figure 3 and Additional file 2, 3, 4). It is interesting that DNA metabolism is most connected in All, followed by DNA/Protein/Amino acid in motile, followed by Protein in Proetobacteria, and finally amino acid in aerobic.

#### Function and hub proteins

**Figure 4:**
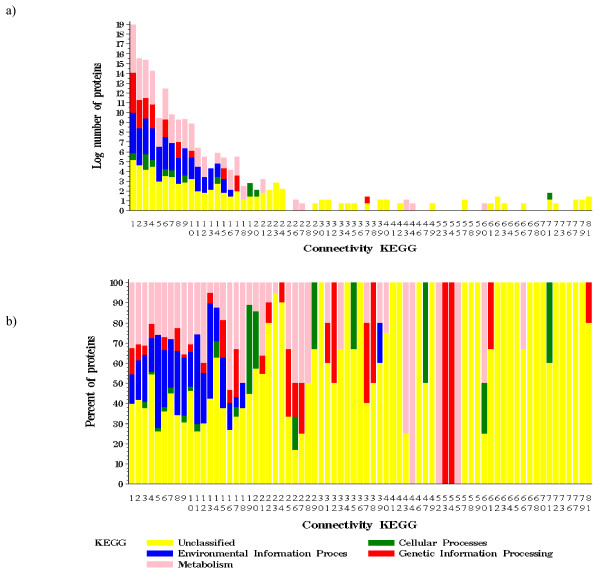
**Functional classification of hub versus non-hub proteins.** Hub proteins are highly connected. a) KEGG connectivity versus log number of proteins. b) KEGG connectivity versus percentage of proteins, normalized within given connectivity.

Power law networks have a few nodes with many connections while the majority of nodes have few links. The nodes with many connections (hubs) are shown to be particularly interesting in a biological context as they can have important roles for drug targets and are crucial in cell viability, among other traits [24]. Intriguingly, in the All network, many of the hubs (connectivity > 19) had no functional classification in any annotation source (Table 3, Figure 4, Additional file 5, 6). Maslov and Sneppen argue that hub proteins tend not to interact with other hub proteins in yeast, but rather prefer to interact with sparsely connected proteins [26]. This assertion was not true in the bacterial co-conservation networks. In bacterial co-conservation networks, hubs tended to interact with other hubs. For example in the All network, of 3,943 interactions (173 hub proteins) where at least one partner was a hub, only 24% (951 interactions) were between a hub and a non-hub while 76% (2,992 interactions) were between two hubs. Hubs were defined as the top 10% of highly connected proteins; the same pattern holds when the hubs were defined to be the top 20% of highly connected proteins (connectivity > 9). For hubs defined as the top 10%, hub-hub connectivity was high in the Motile (70% for connectivity > 44), Proteobacteria (66% for connectivity > 89) and Aerobic (57% for connectivity > 13) networks. Although beyond the scope of this work, it would be interesting to better understand why bacterial hub nodes are highly interconnected and what effect on network properties such interconnectedness confers. Our interest is how connectivity information improves function prediction.

**Table 3:** Connectivity of classified versus unclassified proteins in the co-conserved protein-protein interaction network according to different sources of annotations.

	KEGG classified/unclassified				COG classified/unclassified				TIGR classified/unclassified			
	
	All	Motile	Proteo	Aerobic	All	Motile	Proteo	Aerobic	All	Motile	Proteo	Aerobic
Number of proteins	970/730	975/1015	991/1081	791/619	1271/429	1379/611	1382/690	1121/289	999/701	1013/977	1045/1027	840/570
Mean connectivity	6.47/10.53	13.12/20.70	13.70/31.92	5.44/5.40	6.95/11.95	13.50/24.85	13.53/42.58	5.49/5.159	7.36/9.43	13.88/20.21	13.21/33.38	5.83/4.82
Std connectivity	9.29/16.34	21.14/32.48	27.83/51.65	5.24/5.60	9.63/19.30	21.09/37.69	26.29/59.96	5.20/6.21	10.47/15.78	21.61/32.66	24.33/53.99	5.44/5.34
Max connectivity	81/81	135/147	199/199	35/35	81/81	147/147	199/199	35/35	81/81	142/147	199/199	35/35

### Using topological features to assign function to uncharacterized proteins

Based on the above observations, we hypothesized that specific aspects (i.e. connectivity) of network topology could be used to improve function prediction of uncharacterized proteins. A popular approach to function prediction is to use the most frequently occurring function among the neighbors of an uncharacterized protein [42]. One problem with this approach is that many neighbors of unclassified proteins tend to also be unclassified. For example, combining functional information from KEGG [43], COG [39] and TIGR [44], 41% of the total number of interactions (2,863/6,987 interactions) contained at least one unclassified protein (546 proteins, 233 unclassified) in the All network. In 67% of the 2,863 interactions, one partner was unclassified (479 proteins, 166 unclassified), while in the remaining 33% both proteins in the pair were unclassified (184 unclassified proteins).

Importantly, the majority (60%) of neighbors of the unclassified proteins were also unclassified using KEGG. Based on our observations regarding the topology of the co-conservation network described above, we hypothesized that extending the function prediction strategy to include the majority function assignment of a cluster, rather than just the neighbors of a protein, would improve prediction.

The accuracy of this prediction strategy was validated by determining the average percentage of proteins which were assigned the majority function within their cluster. The percentage value that defines the majority was calculated for each cluster and averaged over all clusters that had at least three proteins and at least two were classified. On average, 93% of classified proteins in a cluster were assigned the KEGG function which was the majority assignment in the cluster. This means taking the majority vote of the cluster had an average prediction error of 7%. Figure 2 further indicates how the prediction accuracy is influenced by cluster size. Although 16% of the clusters have no characterized proteins at all, using the entire cluster to predict function of unclassified proteins is a large improvement over using immediate neighbors since 60% of those were unclassified using KEGG.

**Figure 5:**
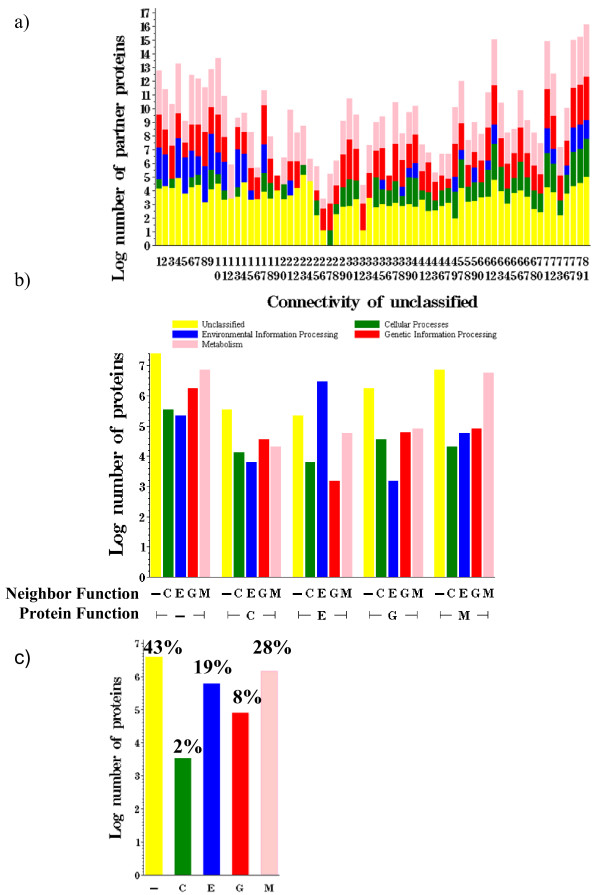
Functional classification using KEGG a) classification of neighbors of unclassified proteins, separated by connectivity of the unclassified protein b) histogram of classification of neighbors given classification of protein c) histogram of classification of all proteins.

The distribution of functional assignment of neighbors of an unclassified protein also varied based on the connectivity of the protein (Figure 5a). There was a higher probability that an unknown protein connected to an Environmental or Metabolism protein if the connectivity was low (k = 1–15) whereas it was improbable to connect to an Environmental protein for connectivities of 22–34. In fact, the distributions suggested the unknown proteins of degree 12 and 24 might be Metabolism proteins, as only that category appeared among their notated neighbors. For connectivity 12, there were 13 classified proteins; 12 are Metabolism proteins and 1 was involved in Genetic information processing. For the 5 classified proteins of connectivity 24, all of them were Metabolism proteins.

Predicting function based on the majority assignment of immediate neighbors or entire cluster both rely on the assumption that like interacts with like. However, it is interesting to note that this may not be valid, as suggested by the distributions of functional assignments of neighbors of characterized proteins (Figure 5b). Though the accuracy of the distributions was confounded by the strong presence of unclassified proteins, they suggested Cellular Process proteins preferentially interacted with Genetic Information Processing proteins. Moreover, Genetic proteins slightly preferred to partner with Metabolism proteins, with a small bias against interacting with Environmental proteins. For Environmental Information Processing and Metabolism proteins, we see the expected behavior of interactions between proteins of the same function. This suggests that function prediction based on majority function will be more accurate for proteins whose true function is either of the latter and less accurate for the two former categories. This claim is investigated below.

#### Evaluating predictions based on topological properties

Of the 730 unclassified proteins (using KEGG) in the All co-conservation network, 369 proteins had no annotated neighbors, while 271 proteins were contained in a cluster containing no annotated neighbors. This means that in the former case, a function prediction algorithm based on immediate neighbors would fail, while in the latter case, a prediction algorithm which uses the entire cluster would fail. For cases such as these in which nearby annotations are not available, the distributions of Figure 5 suggest ways in which topological information might instead be exploited in function prediction algorithms.

Based on the observations detailed above, new predictions strategies were created to incorporate the connectivity of the protein and the differential preference of interaction based on the functions assigned to a protein pair. The contribution of each type and the combination of topological information was evaluated using a cross validation scheme where 10% of the 1,700 classified proteins in the All network with at least one classified neighbor were taken at random as the test set, their functions hidden and predicted from quantities computed on the remaining 90% of classified proteins (training set). From this training set, distributions of node connectivity, function within a cluster, and function among immediate neighbors in a cluster were calculated.

**Figure 6:**
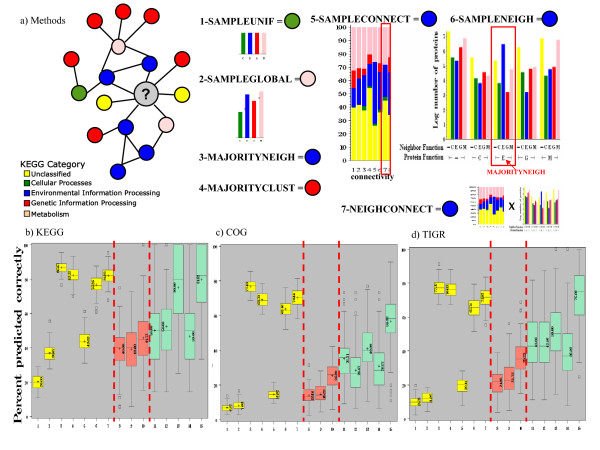
**Cross validation results for function prediction methods in the All network.** a) Illustration of Methods 1–7 on example cluster, b-d) Boxplots of the percentage of correct function assignment, over 100 random partitions into training (90%) and test (10%) sets, using the following prediction methods: 1- SAMPLEUNIF (sampled uniformly at random), 2- SAMPLEGLOBAL (sampled based on the global distribution), 3- MAJORITYNEIGH (the majority assignment to immediate neighbors), 4- MAJORITYCLUST (the majority assignment within the cluster), 5- SAMPLECONNECT (sampled from the distribution of functions for a given connectivity), Method 6- SAMPLENEIGH (sampled from preference bias for non-like pairs by determining the majority function of immediate neighbors, selecting proteins in the cluster with that function and sampling from the distribution of functions assigned to their neighbors), and 7- NEIGHCONNECT (sampled from the combined distribution of SAMPLENEIGH and SAMPLECONNECT). Methods 8–10 apply Methods 5–7 to proteins incorrectly predicted by Method 4-MAJORITYCLUST while Methods 11–15 apply Methods 3–7 to hub proteins. a) KEGG b) COG c) TIGR.

Figure 6 shows boxplots of the percentage of correct function assignment, over 100 random partitions into training and test sets, using the following prediction methods (described more fully in the Methods): 1-SAMPLEUNIF (sampled uniformly at random), 2-SAMPLEGLOBAL (sampled based on the global distribution of functions), 3-MAJORITYNEIGH (the majority functional assignment to immediate neighbors), 4-MAJORITYCLUST (the majority functional assignment within the cluster), 5-SAMPLECONNECT (sampled from the distribution of functions for a given connectivity), 6-SAMPLENEIGH (sampled from preference bias for non-like pairs by determining the majority function of immediate neighbors, selecting proteins in the cluster with that function and sampling from the distribution of functions assigned to their neighbors) and 7-NEIGHCONNECT (sampled from the combined distribution of SAMPLENEIGH and SAMPLECONNECT) (Figure 6a). Methods 8–10 apply Methods 5–7 to proteins incorrectly predicted by Method 4-MAJORITYCLUST while Methods 11–15 apply Methods 3–7 to hub proteins (top 10%, connectivity > 19).

Results in Figure 6 show that the two baseline prediction methods, 1-SAMPLEUNIF and 2-SAMPLEGLOBAL, which do not incorporate topological information had the poorest performances regardless of the annotation source used. A significant improvement (p < 0.001 for all three annotation sources) over these baseline algorithms was seen using connectivity information alone (5-SAMPLECONNECT). Using KEGG, nearly 44% of the proteins were classified correctly using just connectivity information. These results on classified proteins argue that despite being a simple topological characterization, connectivity could be particularly useful for unclassified proteins residing in clusters with no functional information, since majority-based methods can not be applied.

Methods which can incorporate functional information of the cluster in any form (Methods 3–4, 6–7) show substantial improvement over the baselines. Though the 3-MAJORITYNEIGH method outperformed the 4-MAJORITYCLUST method, the prediction task was made easier in the evaluation than would be the case for true unclassified proteins; the requirement that each test protein be connected to at least one other (true) classified protein ensured that there were likely labeled proteins in the immediate neighborhood from which to predict (proteins which had no classified neighbors as a result of creating the test set were not considered in the percent correct count).

The combination (7-NEIGHCONNECT) of connectivity information (5-SAMPLECONNECT) and preference bias for non-like pairs (6-SAMPLENEIGH) showed a significant improvement over preference bias alone for COG (p < 0.0001) and KEGG (p < 0.0001). Moreover, 7-NEIGHCONNECT showed no statistically significant difference in performance from 4-MAJORITYCLUST for KEGG and COG (p < 0.0001 for TIGR).

These results indicated that choosing the majority function of the cluster or immediate neighborhood were better methods on average. However, when the set of proteins was divided into those whose function was predicted correctly or incorrectly using 4-MAJORITYCLUST, most incorrect predictions were for proteins from clusters with a heterogeneous, almost uniform, distribution of function within the cluster. Of the proteins whose function was predicted correctly by 4- MAJORITYCLUST, only 30% resided in clusters with more than one function, compared to 100% for the set of incorrectly predicted proteins.

Methods 8–10 show the value of using connectivity information or preference bias of interaction when the function of the protein is not the majority function of the cluster. The results show the benefit of using connectivity and preference bias information, allowing up to 44% correct prediction (KEGG) for the set of proteins completely missed by one of the best methods, 4-MAJORITYCLUST. Though the average value was similar for 2-SAMPLEGLOBAL and 5-SAMPLECONNECT on all proteins, SAMPLEGLOBAL performed much worse than SAMPLECONNECT on this set of proteins (average for SAMPLEGLOBAL was 10% that of the average of SAMPLECONNECT, data not shown). Interestingly, the average connectivity of this set of incorrectly predicted proteins was high (KEGG 18.53, COG 31.65 and TIGR 28.19), suggesting that using topological information in predictions might be most useful for hub proteins.

Methods 11–15 test the prediction accuracy on the hub proteins, showing dramatic drops in performance for MAJORITYNEIGH (3 vs.11) and MAJORITYCLUST (4 vs. 12). In contrast, using connectivity information within SAMPLECONNECT (5 vs. 13) showed a sharp increase in performance for the set of hubs. The SAMPLENEIGH method (14), based on neighbor information, suffered from noise introduced from the high connectivity, yet when combined with connectivity information (NEIGHCONNECT, 7 and 15) allowed further improvement over connectivity information alone, even for the hubs. Moreover, for the highly connected proteins, the topological based methods SAMPLECONNECT and NEIGHCONNECT outperformed the majority based methods, MAJORITYNEIGH and MAJORITYCLUST.

Together these results suggest an improved function prediction algorithm for truly unclassified proteins, based on the topological properties examined here. When there is a single function assigned to the classified proteins in the cluster, use the majority function of the cluster (4-MAJORITYCLUST) for the uncharacterized protein. Homogeneous neighbors and clusters are generally found for low connectivity proteins. When there is more than one function represented by classified proteins in the cluster, use a combination of connectivity and preference bias information (7-NEIGHCONNECT) for prediction. This situation generally occurs for highly connected proteins.

Overall, our results show connectivity is particularly useful for characterization of unclassified proteins residing in clusters where majority based methods either cannot be applied (i.e. clusters lacking functional information) or would likely fail (i.e. highly connected proteins).

### Function predictions

In the artificial situation represented by our cross validation study, results showed that the majority based methods were effective for proteins whose immediate neighbors or cluster members were generally assigned a single function. For the true unclassified proteins, Additional file 7 shows the majority cluster method applied to the smaller clusters containing at least one unclassified protein and at least one classified protein using any annotation source. Of the 96 proteins in these 22 clusters, 38 proteins were unclassified by any annotation source. Comparing our predictions to a later release of the COG database which provided new annotations for 14 of the 38 proteins, we predicted 13 out of the 14 correctly using the majority cluster method.

## Conclusion

Bacterial co-conservation networks share some topological properties with yeast protein-protein interaction and other biological networks, but differ in important aspects. Like in yeast PPI networks, highly connected nodes are related to essential functions, and the co-conserved protein-protein interaction network appears to be scale free.

It is unclear if the lack of variation observed in the remaining classes represents a true biological phenomenon or a limitation of resolution. There is no difference between the connectivity distributions of complexed and non-complexed proteins in our data to justify the use of connectivity for complex prediction. In this respect, co-conservation networks appear to be distinct from physical interaction networks. However connectivity in our networks does appear to be a reliable predictor for essentiality, in congruence with previous findings [24,35,36,45].

We took advantage of several properties of the network to infer function for several of the uncharacterized proteins in *E. coli *K12 (Additional file 7). The analysis of functional assignment for individual proteins and all protein pairs (Figure 4 and Figure 5) showed that the frequency of interaction between proteins depended on the function of each partner and on connectivity. An interesting future direction would be to incorporate these global observations within a function prediction algorithm and test the accuracy of using connectivity and neighbor function assignment information together to refine the likelihood of assigning a given function to an unknown protein.

## Methods

### Bacteria selection

At the time of this implementation (June 2006), 268 complete microbial genomes were available through the National Center for Biotechnology Information (NCBI) and were downloaded from their ftp site [46]. *E. coli *K12 was selected as the target since a well curated dataset of protein functions was available [47] and substantial experimental data existed for this bacteria. Phenotypic information such as motility and oxygen requirement was generated manually from available data at NCBI. Several different reference genomes were used in our system: 1) All the fully sequenced bacteria available at NCBI (All (268 bacteria)); 2) selecting based on Motility (Motile (104 bacteria)); 3) selecting all proteobacterial species (Proteobacteria (130 bacteria)); and 4) selecting based on oxygen requirement (Aerobic (91 bacteria)).

### Creating phylogenetic profiles matrix

Pairwise one-against-all BLAST searches were performed to identify all proteins in the set of reference organisms homologous to proteins in the target. For each protein i of the target organism *E. coli *K12, the BLAST E-value of the top scoring sequence alignment between protein i and all the proteins of each reference genome j was assign to E_ij_. The phylogenetic profile was constructed as a vector with elements P_ij_, where P_ij _= 1 if a homolog exists (E_ij _< 10^-5^) for the same protein in genome j, otherwise P_ij _= 0.

We eliminated the proteins that appear in more than 90% and less than 10% of organisms before measuring profile similarities (as described below) since proteins that appear in almost all organisms are likely to fall in many functional categories (thereby adding unnecessary noise to the prediction task) and proteins that appear in few organisms are likely to be organism specific. Eliminating these proteins avoid erroneously asserting interactions among proteins whose profiles artificially have high correlation due to an overabundance of zeros or ones, rather than any real biological significance. Previous work [8] characterized the discarded proteins based on COG classifications and the majority of proteins that appeared in more than 90% of the reference genomes were involved in translation, ribosomal structure and biogenesis. Additional file 8 and Additional file 2, 3, 4 provide the complementary topological analysis and comparison of connectivity when the proteins appearing in more than 90% or less than 10% of the reference genomes were not removed.

### Measuring profile similarities

Given a set of phylogenetic profiles, the similarity between any pair of proteins can be calculated using the Pearson correlation coefficient. Similarity between two profiles for protein X and Y using Pearson correlation coefficient was calculated as in [1,4,48]

$$r=\frac{f_{Z}-f_{X}f_{Y}}{\sqrt{\left(f_{X}-f_{X}^{2})(f_{Y}-f_{Y}^{2}\right)}}$$

where

*f*_*X *_= *(I/N)*, *f*_*Y *_= *(J/N)*, and *f*_*Z *_= *(K/N)*.

*N *is the number of organisms in the reference set, *I *is the sum of P_Xj _over all reference genomes j, *J *is the sum of P_Yj _over j, and *K *is the sum over the subset of genomes that contain homologs of both X and Y.

### Generating the interaction networks

Networks were created and presented as graphs in which each protein was represented as a node and an interaction between proteins was represented by an edge. In the co-conservation networks, an edge existed between a pair of proteins whose phylogenetic profiles similarity score exceeded a given threshold (> 0.80). The physical protein-protein interaction network was created by extracting all interactions available for *E. coli *from the Database of Interacting Proteins (DIP) [37], downloaded 7 July 2007. For separation of connected components of the network and building the clusters of proteins, breadth-first search graph algorithms were used. Network graphs were visualized using Cytoscape [49] an open-source, platform-independent environment software.

### Functional classification

The functional annotations of *E. coli *K12 proteins were extracted from four databases: Clusters of Orthologous Groups of proteins (COG) at NCBI [39] (downloaded 1/5/2007), KEGG [38] (downloaded 1/5/2007), TIGR [40] (downloaded 1/5/2007) and EcoCyc [47] (version 10.5). *E. coli *protein complex data was also extracted from EcoCyc. Essential proteins were extracted from DEG database [50]. KEGG classified proteins into four functional categories: Metabolism (Carbohydrate Metabolism, Energy Metabolism, Lipid Metabolism, Nucleotide Metabolism, Amino Acid Metabolism, Metabolism of Other Amino Acids, Glycan Biosynthesis and Metabolism, Biosynthesis of Polypeptides and Non ribosomal Pept, Metabolism of Cofactors and Vitamins, Biosynthesis of Secondary Metabolites and Xenobiotics Biodegradation and Metabolism); Genetic Information Processing (Transcription, Translation, Folding Sorting and Degradation and Replication and Repair); Environmental Information Processing (Membrane Transport, Signal Transduction and Signaling Molecules and Interaction); and Cellular Processes (Cell Motility and Cell Growth and Death) [38]. Since TIGR and COG classify proteins into 15 and 18 categories respectfully, these categories were manually aligned to roughly correspond to the four KEGG categories for comparison (see Figure 2).

### Analyzing the topology of the network

The degree of a node in a graph is the number of edges connected to that node and proteins that are joined by an edge are said to be neighbors. The clustering coefficient C indicates the degree to which k neighbors of a particular node are connected to each other. Let *k*_*i *_be the number of neighbors of node i and *n*_*i *_be the number of edges in the network that exist among the neighbors of i. The clustering coefficient [51] of node i is given as

*C*_*i *_= *2 n*_*i*_/*(k*_i _* *(k*_*i*_*-1))*.

Then the average clustering coefficient was calculated by averaging C_i _over all nodes i.

The connectivity distribution P(k), i.e., the probability that a protein interacts with other proteins, was evaluated empirically. The regression coefficient r between log (P (k)) and log (k) could then be calculated. The exponent of the power law distribution γ was estimated from the observed distribution, as the slope of the line log (P (k)) versus log (k). The topology in relation to the function was validated from available information in COG [39], TIGR [40], KEGG [38] and EcoCyc [47] sources. The diameter and average shortest path of the network was calculated using a breath-first search algorithm.

### Defining hubs in network

As connectivity scales with network topology, we did not assign a static degree to define the hubs. The hubs in the network were defined to be the top 10% of the most connected nodes in our network, corresponding to a connectivity exceeding 19 in the All network.

### Determining statistical significance

In Table 2, p-values in Table 2 were calculated using non-parametric Wilcoxon RankSum test in SAS [52].

### Function prediction cross-validation

From the 1,700 proteins in the All co-conservation network, the set of classified proteins with at least one classified neighbor (910, 1321 and 921 respectively according to KEGG, COG and TIGR) was divided uniformly at random into training (90%) and testing (10%) sets. The function of the proteins in the training set were hidden and predicted from methods applied to the training set, and the percentage of correctly predicted proteins was calculated over 100 cross validation splits. Using the training set, the distribution of functional assignment versus connectivity was calculated for all classified training proteins. Additionally, for each cluster, the distribution of the number of interactions between proteins assigned with each pair of functions was calculated. For each distribution, predictions based on sampling the function ignored the count for unclassified proteins, renormalizing among the remaining categories. The following prediction methods were used:

SAMPLEUNIF: predicted function is sampled uniformly at random from the set of categories (KEGG 4 categories, COG 18 categories, TIGR 15 categories)

SAMPLEGLOBAL: predicted function is sampled based on the global distribution of known function among all proteins in the training set (similar to Figure 5c yet calculated on the training set only)

MAJORITYNEIGH: predicted function is the majority assignment to immediate neighbors (ties are broken randomly)

MAJORITYCLUST: predicted function is the majority assignment within the cluster

SAMPLECONNECT: predicted function is sampled from the distribution of functions for given connectivity (similar to Figure 5 yet calculated on the training set only)

SAMPLENEIGH: predicted function is based on first determining the majority function of the immediate neighbors, examining all protein pairs in the cluster involving proteins with that function and sampling from the distribution of functional assignments for the other partners of the pairs (similar to Figure 5b where the function indexed by Protein is the majority function of immediate neighbors and Neighbor refers to partners of all proteins in the cluster with that function)

NEIGHCONNECT: predicted function is sampled from the combined distribution of SAMPLENEIGH and SAMPLECONNECT, calculated by multiplying the two distributions category-wise and then renormalizing across categories.

## Availability

Data are available upon request.

## Authors' contributions

AK–F conceived of the project and implemented the methods. SML and AK–F analyzed the data. The manuscript was written by AK–F and SML and edited by RTG and LEH. RTG oversaw all biological aspects of the work and LEH supervised the computational aspects.

## Supplementary Material

Additional file 1 Co-conserved protein-protein interaction network of *E. coli *K12 using different reference genome sets. The co-conserved protein-protein interaction network of *E. coli *K12 using different reference genome sets. a) All b) Motile c) Proteobacteria d) Aerobic. Center plots show connectivity distribution of co-conserved protein-protein interactions: connectivity (k) versus p(k), and log connectivity (k) versus log p(k). Click here for file

Additional file 2 Average connectivity of each functional category in networks using different reference sets and using KEGG annotation. The average connectivity of each functional category in networks with and without removing proteins appearing in more than 90% or less than 10% of organisms using different reference sets and using KEGG annotation. Click here for file

Additional file 3 Average connectivity of each functional category in networks using different reference sets and using COG annotation. The average connectivity of each functional category in networks with and without removing proteins appearing in more than 90% or less than 10% of organisms using different reference sets and using COG annotation. COG functional categories and subcategories are: Poorly characterized [Not classified (-)]; Information storage and processing [Translation, ribosomal structure and biogenesis (J); Transcription (K); DNA replication, recombination and repair(L)]; Cellular processes [Cell division and chromosome partitioning (D); Posttranslational modification, protein turnover, chaperones (O); Cell envelope biogenesis, outer membrane (M); Cell motility and secretion (N); Inorganic ion transport and metabolism(P); Signal transduction mechanism (T); Intracellular trafficking, secretion, and vesicular transport (U); Defense mechanisms (V)]; Metabolism [Energy production and conversion (C); Carbohydrate transport and metabolism (G); Amino acid transport and metabolism (E); Nucleotide transport and metabolism (F); Coenzyme metabolism (I); Lipid metabolism (H); Secondary metabolites biosynthesis, transport and catabolism (Q)]. Click here for file

Additional file 4 Average connectivity of each functional category in networks using different reference sets and using TIGR annotation. The average connectivity of each functional category in networks with and without removing proteins appearing in more than 90% or less than 10% of organisms using different reference sets and using TIGR annotation. Click here for file

Additional file 5 Functional classification of hub versus non-hub proteins using COG. Functional classification of hub versus non-hub proteins. Hub proteins are highly connected. a) COG connectivity versus log number of proteins. b) COG connectivity versus percentage of proteins, normalized within given connectivity. Click here for file

Additional file 6 Functional classification of hub versus non-hub proteins using TIGR. Functional classification of hub versus non-hub proteins. Hub proteins are highly connected. a) TIGR connectivity versus log number of proteins. b) TIGR connectivity versus percentage of proteins, normalized within given connectivity. Click here for file

Additional file 7 Function prediction in clusters containing at least 1 and maximum 10 proteins using different source of annotations. Function prediction in clusters containing at least 1 and maximum 10 proteins with at least one unclassified proteins across all sources and at least one source of annotation. KEGG version downloaded 1/5/2007, COG version downloaded 1/5/2007, TIGR version downloaded 1/5/2007. Predicted function column lists putative function based on annotated cluster members. As partial validation of the prediction, more recent annotations from EcoCyc version downloaded 1/5/2007 and a later version of COG downloaded 1/7/2007 (New COG) are shown. Click here for file

Additional file 8 Topological analysis of the networks using different reference sets. Topological analysis of the networks without removing proteins appearing in more than 90% or less than 10% of organisms using different reference sets. Click here for file
