# Supplementary figures and images for: The topology of the bacterial co-conserved protein network and its implications for predicting protein function
Source: BMC Genomics. 2008 Jun 30;9:313. doi: 10.1186/1471-2164-9-313 (PMC2488357; doi:10.1186/1471-2164-9-313)

a) All

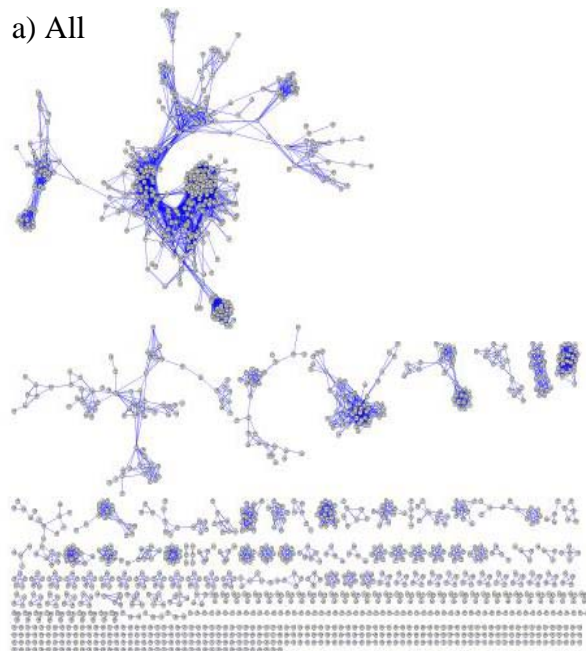

c) Proteobacteria

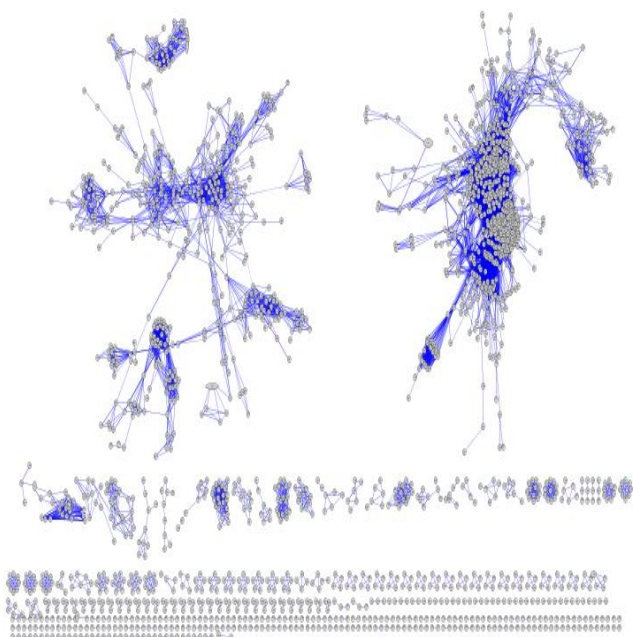

b) Motile

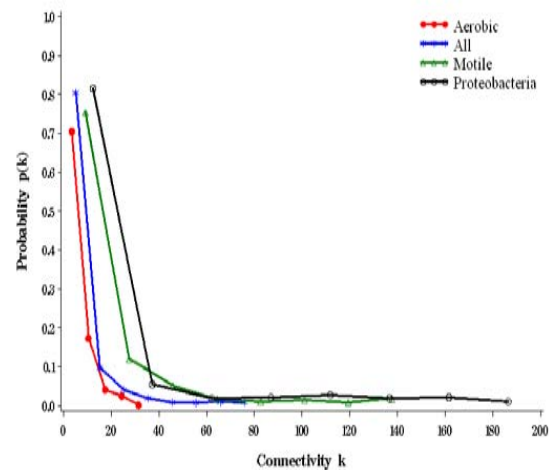

d) Aerobic

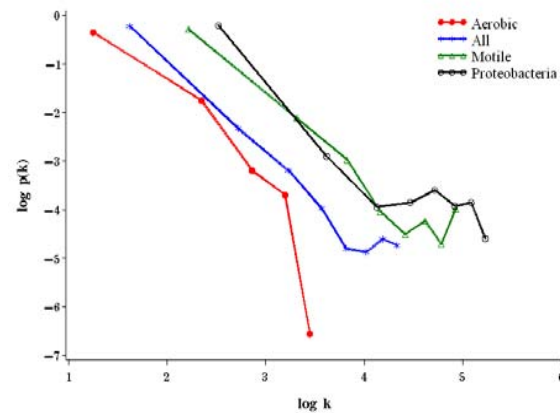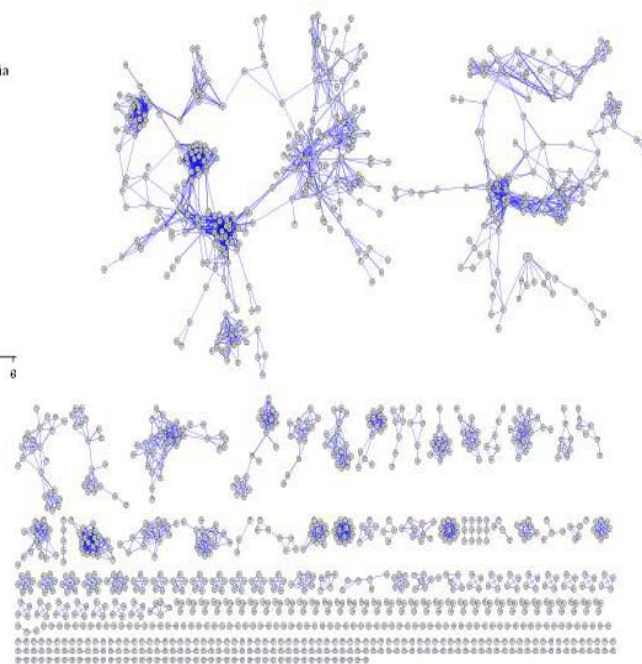

Supplement: Additional file 1 — Co-conserved protein-protein interaction network of E. coli K12 using different reference genome sets. The co-conserved protein-protein interaction network of E. coli K12 using different reference genome sets. a) All b) Motile c) Proteobacteria d) Aerobic. Center plots show connectivity distribution of co-conserved protein-protein interactions: connectivity (k) versus p(k), and log connectivity (k) versus log p(k). [file 1471-2164-9-313-S1.pdf]

a)

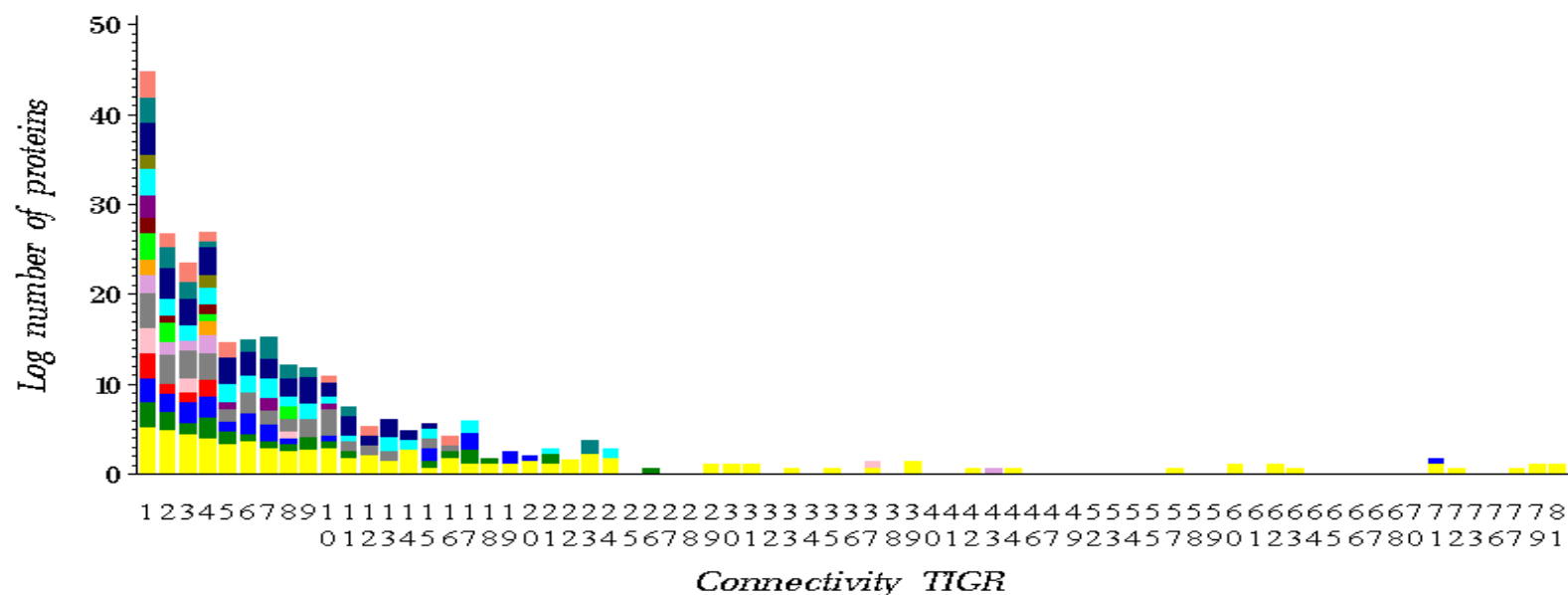

b)

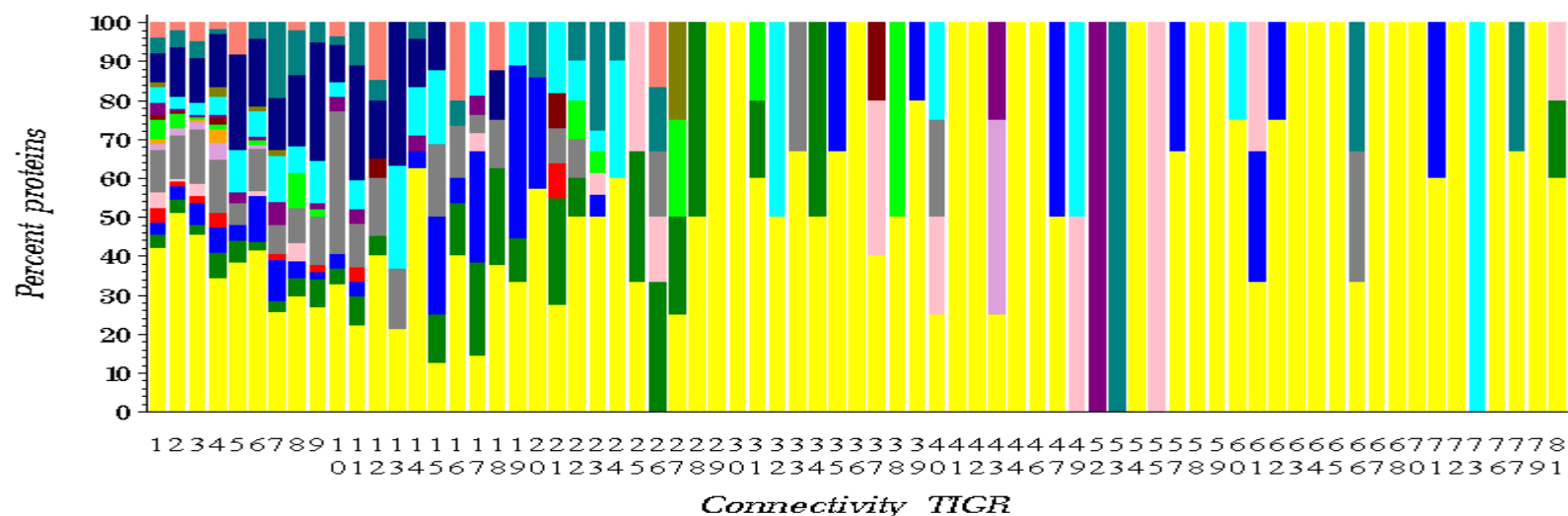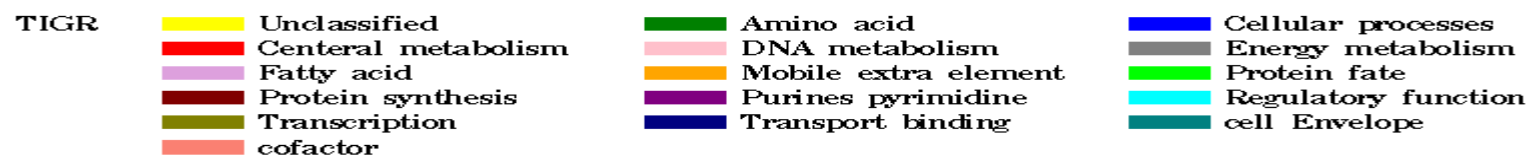

Supplement: Additional file 6 — Functional classification of hub versus non-hub proteins using TIGR. Functional classification of hub versus non-hub proteins. Hub proteins are highly connected. a) TIGR connectivity versus log number of proteins. b) TIGR connectivity versus percentage of proteins, normalized within given connectivity. [file 1471-2164-9-313-S6.pdf]
