# Supplementary material for: The topology of the bacterial co-conserved protein network and its implications for predicting protein function
Source: BMC Genomics. 2008 Jun 30;9:313. doi: 10.1186/1471-2164-9-313 (PMC2488357; doi:10.1186/1471-2164-9-313)
Supplement: Additional file 2 — Average connectivity of each functional category in networks using different reference sets and using KEGG annotation. The average connectivity of each functional category in networks with and without removing proteins appearing in more than 90% or less than 10% of organisms using different reference sets and using KEGG annotation. [file 1471-2164-9-313-S2.pdf]

KEGG

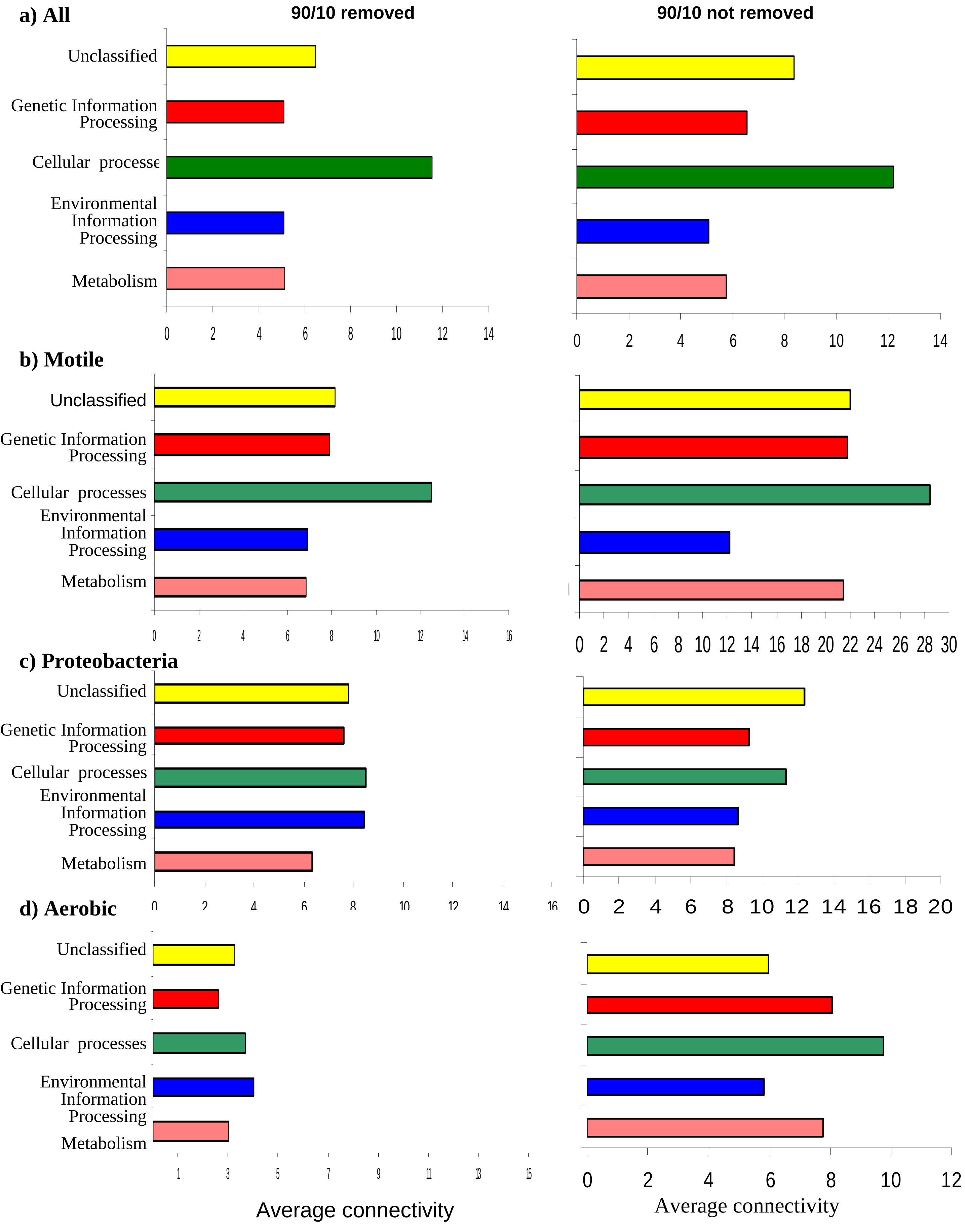
a) All
90/10 removed
90/10 not removed
Unclassified
Genetic Information Processing
Cellular processe
Environmental Information Processing
Metabolism
0 2 4 6 8 10 12 14
0 2 4 6 8 10 12 14
b) Motile
Unclassified
Genetic Information Processing
Cellular processes
Environmental Information Processing
Metabolism
0 2 4 6 8 10 12 14 16
0 2 4 6 8 10 12 14 16 18 20 22 24 26 28 30
c) Proteobacteria
Unclassified
Genetic Information Processing
Cellular processes
Environmental Information Processing
Metabolism
0 2 4 6 8 10 12 14 16
0 2 4 6 8 10 12 14 16 18 20
d) Aerobic
Unclassified
Genetic Information Processing
Cellular processes
Environmental Information Processing
Metabolism
1 3 5 7 9 11 13 15
0 2 4 6 8 10 12
Average connectivity
Average connectivity
