# Supplementary material for: The topology of the bacterial co-conserved protein network and its implications for predicting protein function
Source: BMC Genomics. 2008 Jun 30;9:313. doi: 10.1186/1471-2164-9-313 (PMC2488357; doi:10.1186/1471-2164-9-313)
Supplement: Additional file 3 — Average connectivity of each functional category in networks using different reference sets and using COG annotation. The average connectivity of each functional category in networks with and without removing proteins appearing in more than 90% or less than 10% of organisms using different reference sets and using COG annotation. COG functional categories and subcategories are: Poorly characterized [Not classified (-)]; Information storage and processing [Translation, ribosomal structure and biogenesis (J); Transcription (K); DNA replication, recombination and repair(L)]; Cellular processes [Cell division and chromosome partitioning (D); Posttranslational modification, protein turnover, chaperones (O); Cell envelope biogenesis, outer membrane (M); Cell motility and secretion (N); Inorganic ion transport and metabolism(P); Signal transduction mechanism (T); Intracellular trafficking, secretion, and vesicular transport (U); Defense mechanisms (V)]; Metabolism [Energy production and conversion (C); Carbohydrate transport and metabolism (G); Amino acid transport and metabolism (E); Nucleotide transport and metabolism (F); Coenzyme metabolism (I); Lipid metabolism (H); Secondary metabolites biosynthesis, transport and catabolism (Q)]. [file 1471-2164-9-313-S3.pdf]

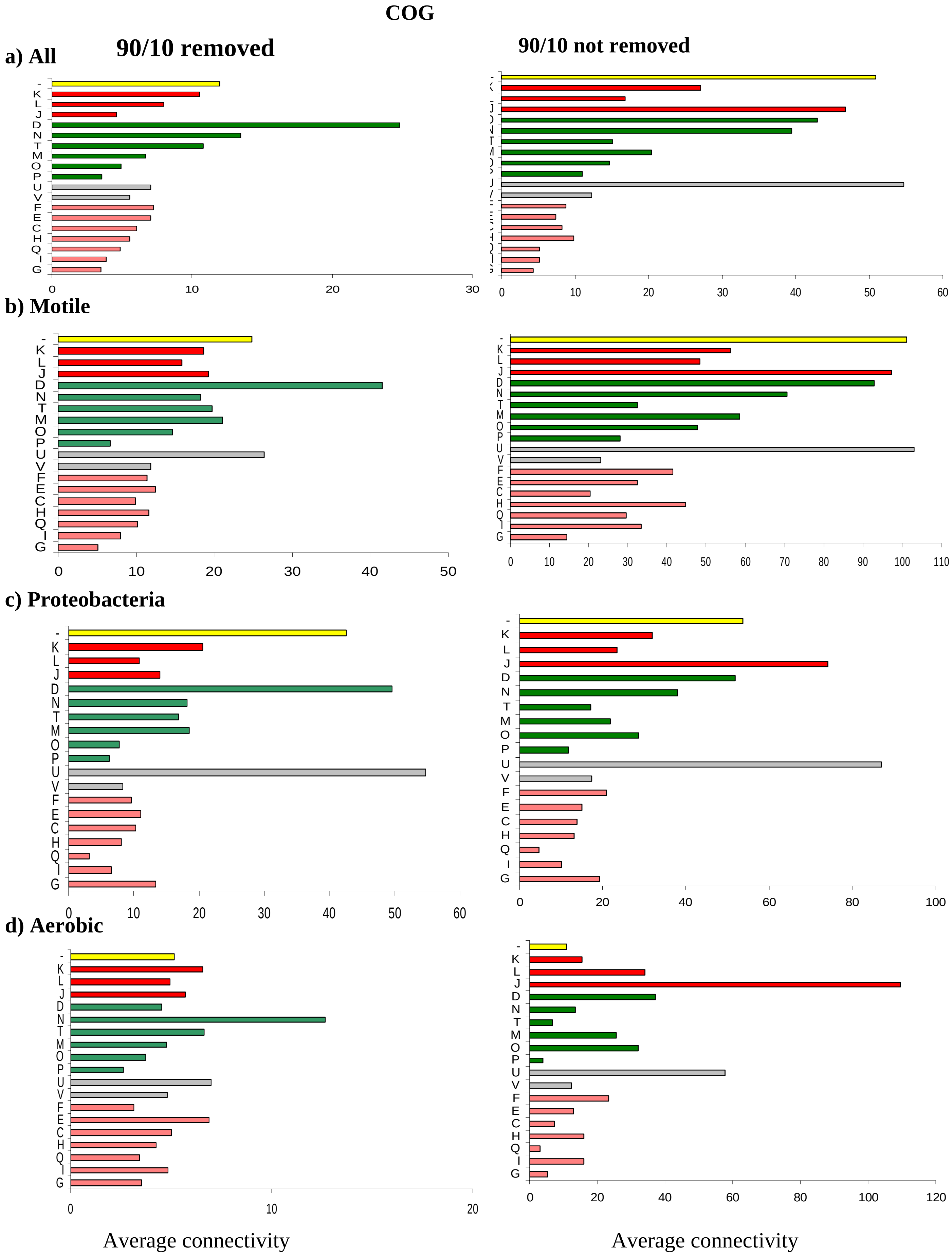
COG
90/10 removed
90/10 not removed
a) All
b) Motile
c) Proteobacteria
d) Aerobic
- K L J D N T M O P U V F E C H Q I G
0 10 20 30
0 10 20 30 40 50 60
0 10 20 30 40 50
0 10 20 30 40 50 60 70 80 90 100 110
0 10 20 30 40 50 60
0 20 40 60 80 100
0 10 20
0 20 40 60 80 100 120
Average connectivity
Average connectivity
