# Supplementary material for: The topology of the bacterial co-conserved protein network and its implications for predicting protein function
Source: BMC Genomics. 2008 Jun 30;9:313. doi: 10.1186/1471-2164-9-313 (PMC2488357; doi:10.1186/1471-2164-9-313)
Supplement: Additional file 4 — Average connectivity of each functional category in networks using different reference sets and using TIGR annotation. The average connectivity of each functional category in networks with and without removing proteins appearing in more than 90% or less than 10% of organisms using different reference sets and using TIGR annotation. [file 1471-2164-9-313-S4.pdf]

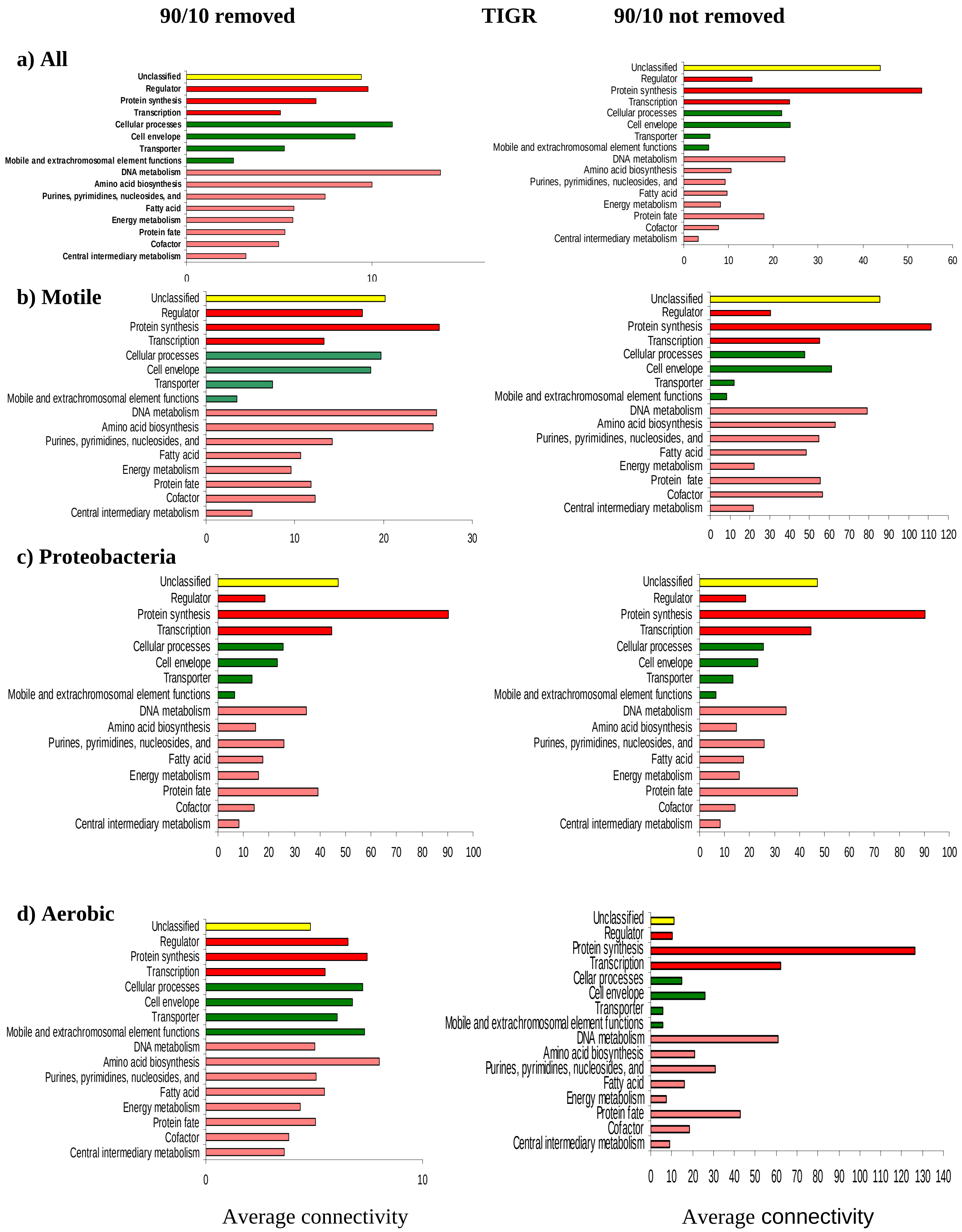
90/10 removed
TIGR
90/10 not removed
a) All
b) Motile
c) Proteobacteria
d) Aerobic
Unclassified
Regulator
Protein synthesis
Transcription
Cellular processes
Cell envelope
Transporter
Mobile and extrachromosomal element functions
DNA metabolism
Amino acid biosynthesis
Purines, pyrimidines, nucleosides, and
Fatty acid
Energy metabolism
Protein fate
Cofactor
Central intermediary metabolism
Cellar processes
Average connectivity
Average connectivity
