# Supplementary material for: The topology of the bacterial co-conserved protein network and its implications for predicting protein function
Source: BMC Genomics. 2008 Jun 30;9:313. doi: 10.1186/1471-2164-9-313 (PMC2488357; doi:10.1186/1471-2164-9-313)
Supplement: Additional file 7 — Function prediction in clusters containing at least 1 and maximum 10 proteins using different source of annotations. Function prediction in clusters containing at least 1 and maximum 10 proteins with at least one unclassified proteins across all sources and at least one source of annotation. KEGG version downloaded 1/5/2007, COG version downloaded 1/5/2007, TIGR version downloaded 1/5/2007. Predicted function column lists putative function based on annotated cluster members. As partial validation of the prediction, more recent annotations from EcoCyc version downloaded 1/5/2007 and a later version of COG downloaded 1/7/2007 (New COG) are shown. [file 1471-2164-9-313-S7.pdf]

NO.	Size	Gene	KEGG function	COG function	TIGR function	Predicted function
1	3	glcF	-	-	-	Energy (EcoCyc central intermediary metabolism, New COG C)
		ykgE	-	Energy production (C)	-	
		glpC	Metabolism	Energy production (C)	Energy metabolism	
2	8	nanR	-	-	-	Regulatory/Transcription (EcoCyc Regulatory, New COG K)
		yieP	-	-	-	Regulatory/Transcription (EcoCyc Regulatory, New COG K)
		dgoR	-	-	Regulatory function	
		lldR	-	Transcription (K)	-	
		exuR	-	Transcription (K)	Regulatory function	
		glcC	-	Transcription (K)	Regulatory function	
		pdhR	-	Transcription (K)	Regulatory function	
		uxuR	-	Transcription (K)	Regulatory function	
3	5	caiC	-	-	-	Metabolism/ Lipid metabolism (EcoCyc amines, New COG I)
		ydiD	-	-	-	Metabolism/ Lipid metabolism (EcoCyc fatty acids, New COG I)
		aas	Metabolism	Lipid metabolism (I)	Fatty acid	
		fadD	Metabolism	Lipid metabolism (I)	Fatty acid	
		entE	Metabolism	Secondary metabolites biosynthesis (Q)	Central metabolism	
4	3	ychQ	-	-	-	Metabolism/ Lipid metabolism (EcoCyc predicted as regulator)
		yhbT	-	Lipid metabolism (I)	-	
		yhbV	-	Posttranslational modification (O)	-	
5	5	ycgR	-	-	-	Cellular processes/Cell motility and secretion (EcoCyc regulator of flagellar motility, New COG M)
		flgA	Cellular processes	Cell motility and secretion (N)	Cellular processes	
		fliL	Cellular processes	Cell motility and secretion (N)	Cellular processes	
		flhC	Environmental Information	-	Cellular processes	
		flhD	Environmental Information	-	Cellular processes	
6	4	yjfR	-	-	-	Carbohydrate transport and metabolism (EcoCyc carbon utilization)
		sgaB	Environmental Information	Carbohydrate transport and metabolism (G)	Energy metabolism	
		sgaU	Metabolism	Carbohydrate transport and metabolism (G)	-	
		sgbU	Metabolism	Carbohydrate transport and metabolism (G)	-	
7	3	ycgB	-	-	-	Signal transduction mechanism
		yeaH	-	-	-	Signal transduction mechanism
		yeaG	-	Signal transduction mechanism (T)	-	
8	3	ydcG	-	-	-	Cell envelop biogenesis (EcoCyc osmotic pressure)
		mdoH	-	Cell envelope biogenesis (M)	-	
		mdoG	-	Inorganic ion transport and metabolism	-	
9	9	yfiD	-	-	-	??? (EcoCyc energy metabolism)
		yjjW	-	Posttranslational modification (O)	-	
		pflC	Genetic Information	-	Protein fate	
		pflA	Genetic Information	Posttranslational modification (O)	Energy metabolism	
		ybiY	Genetic Information	Posttranslational modification (O)	Protein fate	

		tdcE	Metabolism	-	Energy metabolism	
		pflB	Metabolism	Energy production (C)	Energy metabolism	
		pflD	Metabolism	Energy production (C)	Protein fate	
		ybiW	Metabolism	Energy production (C)	Protein fate	
10	4	rpiR	-	-	-	Regulatory/Transcription (EcoCyc regulator, New COG K)
		yfeT	-	Transcription (K)	-	
		yfhH	-	Transcription (K)	-	
		yebK	-	Transcription (K)	Regulatory function	
11	4	yiiL	-	-	-	Metabolism (EcoCyc carbon utilization)
		yjfP	-	-	-	Metabolism
		rhaA	Metabolism	-	-	
		rhaD	Metabolism	Carbohydrate transport and metabolism (G)	Energy metabolism	
12	3	yghU	-	-	-	Posttranslational modification (EcoCyc centeral int. metabolism, New COG O)
		yliJ	-	-	-	Posttranslational modification (New COG O)
		yfcG	-	Posttranslational modification (O)	-	
13	3	yehR	-	-	-	Carbohydrate transport and metabolism
		gatA	Metabolism	Carbohydrate transport and metabolism (G)	-	
		gatB	Metabolism	Carbohydrate transport and metabolism (G)	-	
14	6	ydhC	-	-	-	Carbohydrate transport and metabolism (EcoCyc MFS transporter)
		emrD	-	Carbohydrate transport and metabolism (G)	-	
		bcr	-	Carbohydrate transport and metabolism (G)	Cellular processes	
		cmr	-	Carbohydrate transport and metabolism (G)	Cellular processes	
		yidY	-	Carbohydrate transport and metabolism (G)	Cellular processes	
		yjiO	-	Carbohydrate transport and metabolism (G)	Cellular processes	
15	3	leuO	-	-	-	???? (EcoCyc Transcription, New COG K)
		ybeF	-	-	Energy metabolism	??? (New COG K)
		yidZ	-	Transcription (K)	-	
16	5	yhfR	-	-	-	Regulatory/Transcription (EcoCyc Transcription)
		farR	-	Transcription (K)	-	
		phnF	-	Transcription (K)	-	
		yegW	-	Transcription (K)	-	
		yidP	-	Transcription (K)	-	
17	4	araJ	-	-	-	Carbohydrate transport and metabolism (EcoCyc MFS transporter, New COG G)
		sotB	-	Carbohydrate transport and metabolism (G)	-	
		ydhP	-	Carbohydrate transport and metabolism (G)	-	
		yicM	-	Carbohydrate transport and metabolism (G)	Regulatory function	
18	4	yibP	-	-	-	Cell envelope biogenesis (EcoCyc cell processes, cell division)
		ygeR	-	-	Cell envelope	
		yebA	-	Cell envelope biogenesis (M)	-	
		nlpD	-	Cell envelope biogenesis (M)	Cell envelope	
19	3	yjgK	-	-	-	Carbohydrate transport and metabolism
		yhcH	-	Carbohydrate transport and metabolism (G)	-	
		yiaL	-	Carbohydrate transport and metabolism (G)	-	
20	8	ydhF	-	-	-	Energy production /cofactor

		yeaE	-	-	-	Energy production /cofactor (EcoCyc central int. metabolism ???
		dkgA	-	-	cofactor	
		dkgB	-	-	cofactor	???
		tas	-	Energy production (C)	-	
		yajO	-	Energy production (C)	-	
		ydjG	-	Energy production (C)	-	
		yghZ	-	Energy production (C)	-	
21	3	ygcN	-	-	-	Energy production (New COG C)
		fixC	-	Energy production (C)	-	
		ydiS	-	Energy production (C)	-	
22	3	ybgL	-	-	-	Amino acid transport and metabolism
		ybgJ	-	Amino acid transport and metabolism (E)	-	
		ybgK	-	Amino acid transport and metabolism (E)	-	
