# Supplementary material for: The topology of the bacterial co-conserved protein network and its implications for predicting protein function
Source: BMC Genomics. 2008 Jun 30;9:313. doi: 10.1186/1471-2164-9-313 (PMC2488357; doi:10.1186/1471-2164-9-313)
Supplement: Additional file 8 — Topological analysis of the networks using different reference sets. Topological analysis of the networks without removing proteins appearing in more than 90% or less than 10% of organisms using different reference sets. [file 1471-2164-9-313-S8.pdf]

	All	Motile	Proteo	Aerobic
Number of interactions (edges)	41,331	102,239	54,130	21,846
Number of proteins (nodes)	2,721	3,098	2,988	2,244
Log-log correlation (r)	0.87	0.75	0.77	0.58
Power law exponent (γ)	1.17	1.12	1.18	1.08
Average clustering coefficient (c)	0.80	0.76	0.76	0.80
Connectivity average (k)	30.38	66.00	36.22	19.47
Standard deviation of connectivity	52.21	100.00	57.62	44.55
Average shortest path	5.21	9.30	5.94	6.36
Diameter	18	24	25	20
