# Supplementary material for: The topology of the bacterial co-conserved protein network and its implications for predicting protein function
Source: BMC Genomics. 2008 Jun 30;9:313. doi: 10.1186/1471-2164-9-313 (PMC2488357; doi:10.1186/1471-2164-9-313)
Supplement: Additional file 5 — Functional classification of hub versus non-hub proteins using COG. Functional classification of hub versus non-hub proteins. Hub proteins are highly connected. a) COG connectivity versus log number of proteins. b) COG connectivity versus percentage of proteins, normalized within given connectivity. [file 1471-2164-9-313-S5.pdf]

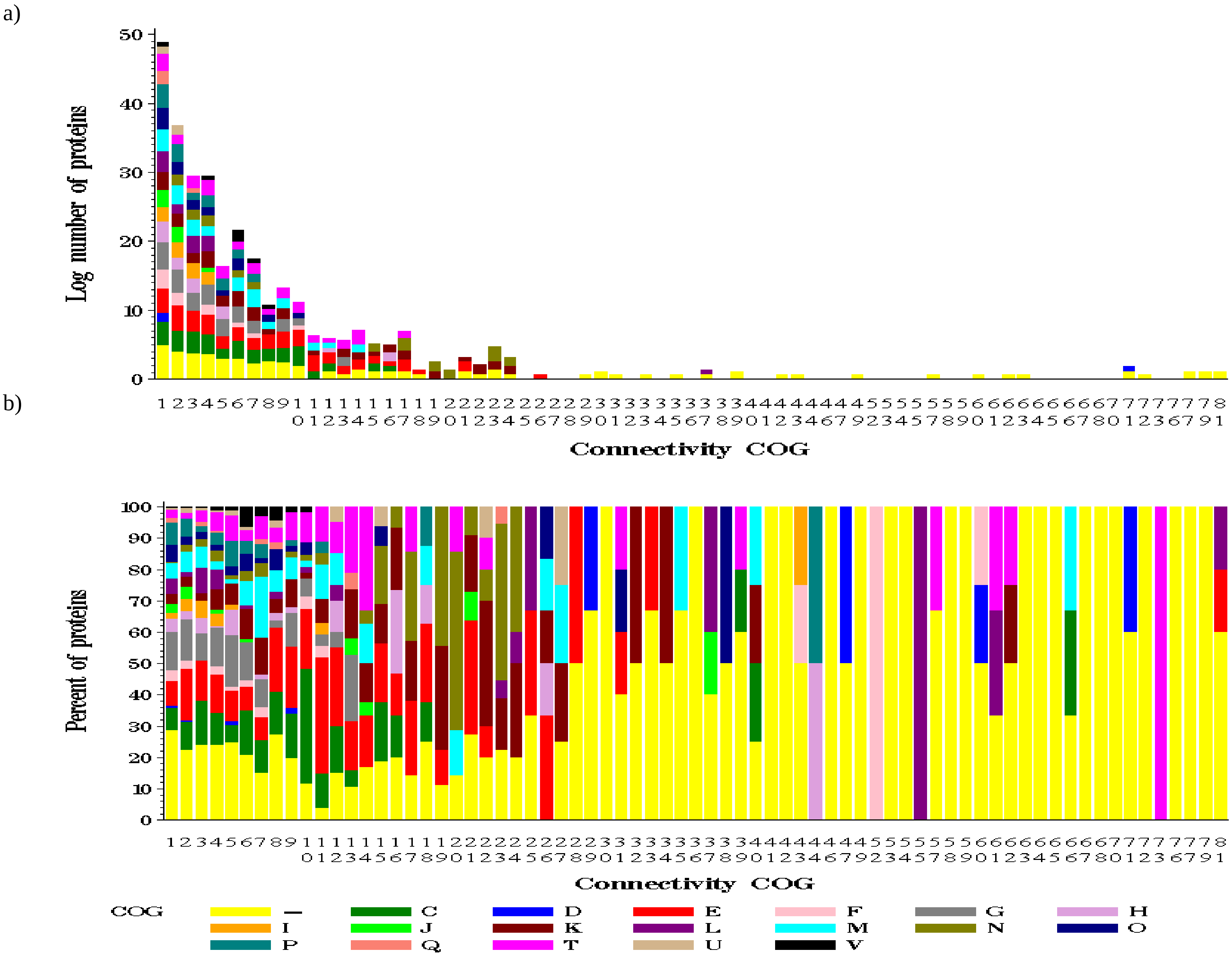
a)
Log number of proteins
0
10
20
30
40
50
Connectivity COG
b)
Percent of proteins
0
10
20
30
40
50
60
70
80
90
100
Connectivity COG
COG
—
C
D
E
F
G
H
I
J
K
L
M
N
O
P
Q
T
U
V
